# Supplementary material for: Modeling glioblastoma heterogeneity as a dynamic network of cell states
Source: Mol Syst Biol. 2021 Sep 16;17(9):e10105. doi: 10.15252/msb.202010105 (PMC8444284; doi:10.15252/msb.202010105)
Supplement: Supplementary file 6 — Source Data for Figure 5 [file MSB-17-e10105-s004.zip › Figure5A_sourcedata/GSEA_3017/nosigdb_stateB.GseaPreranked.1622795021725/SEURAT_CELLCYCLE.html]

Details for gene set SEURAT\_CELLCYCLE[GSEA]

|  || Dataset | state43017 |
| Phenotype | NoPhenotypeAvailable |
| Upregulated in class | na\_pos |
| GeneSet | SEURAT\_CELLCYCLE |
| Enrichment Score (ES) | 0.6174586 |
| Normalized Enrichment Score (NES) | 3.4799976 |
| Nominal p-value | 0.0 |
| FDR q-value | 0.0 |
| FWER p-Value | 0.0 |
Table: GSEA Results Summary

  

Fig 1: Enrichment plot: SEURAT\_CELLCYCLE      
 Profile of the Running ES Score & Positions of GeneSet Members on the Rank Ordered List

  

| PROBE | GENE SYMBOL | GENE\_TITLE | RANK IN GENE LIST | RANK METRIC SCORE | RUNNING ES | CORE ENRICHMENT || 1 | TOP2A |  |  | 3 | 1.079 | 0.0239 | Yes |
| 2 | RRM2 |  |  | 7 | 0.875 | 0.0425 | Yes |
| 3 | UBE2C |  |  | 11 | 0.802 | 0.0592 | Yes |
| 4 | TPX2 |  |  | 12 | 0.801 | 0.0803 | Yes |
| 5 | NUSAP1 |  |  | 13 | 0.801 | 0.1013 | Yes |
| 6 | HMGB2 |  |  | 14 | 0.786 | 0.1219 | Yes |
| 7 | CDCA2 |  |  | 19 | 0.751 | 0.1358 | Yes |
| 8 | TACC3 |  |  | 21 | 0.741 | 0.1538 | Yes |
| 9 | GTSE1 |  |  | 22 | 0.732 | 0.1730 | Yes |
| 10 | HJURP |  |  | 23 | 0.731 | 0.1922 | Yes |
| 11 | KIF23 |  |  | 24 | 0.721 | 0.2111 | Yes |
| 12 | NDC80 |  |  | 27 | 0.695 | 0.2264 | Yes |
| 13 | ATAD2 |  |  | 28 | 0.685 | 0.2444 | Yes |
| 14 | CENPF |  |  | 34 | 0.670 | 0.2547 | Yes |
| 15 | ANLN |  |  | 39 | 0.659 | 0.2662 | Yes |
| 16 | RAD51AP1 |  |  | 40 | 0.657 | 0.2834 | Yes |
| 17 | SMC4 |  |  | 41 | 0.655 | 0.3006 | Yes |
| 18 | CENPE |  |  | 43 | 0.654 | 0.3163 | Yes |
| 19 | NUF2 |  |  | 44 | 0.651 | 0.3334 | Yes |
| 20 | DLGAP5 |  |  | 46 | 0.647 | 0.3489 | Yes |
| 21 | CENPA |  |  | 51 | 0.636 | 0.3598 | Yes |
| 22 | CKAP2 |  |  | 53 | 0.628 | 0.3748 | Yes |
| 23 | AURKA |  |  | 55 | 0.617 | 0.3896 | Yes |
| 24 | CKAP2L |  |  | 59 | 0.611 | 0.4013 | Yes |
| 25 | TYMS |  |  | 63 | 0.601 | 0.4127 | Yes |
| 26 | KIF2C |  |  | 66 | 0.591 | 0.4253 | Yes |
| 27 | CDK1 |  |  | 69 | 0.585 | 0.4377 | Yes |
| 28 | CKS1B |  |  | 72 | 0.575 | 0.4499 | Yes |
| 29 | CDCA8 |  |  | 82 | 0.562 | 0.4515 | Yes |
| 30 | CKS2 |  |  | 83 | 0.558 | 0.4662 | Yes |
| 31 | ECT2 |  |  | 84 | 0.558 | 0.4808 | Yes |
| 32 | AURKB |  |  | 89 | 0.548 | 0.4894 | Yes |
| 33 | RRM1 |  |  | 103 | 0.520 | 0.4841 | Yes |
| 34 | CDCA3 |  |  | 111 | 0.511 | 0.4874 | Yes |
| 35 | BUB1 |  |  | 113 | 0.508 | 0.4993 | Yes |
| 36 | TUBB4B |  |  | 114 | 0.507 | 0.5125 | Yes |
| 37 | BIRC5 |  |  | 116 | 0.504 | 0.5243 | Yes |
| 38 | CLSPN |  |  | 117 | 0.503 | 0.5375 | Yes |
| 39 | E2F8 |  |  | 125 | 0.493 | 0.5403 | Yes |
| 40 | TMPO |  |  | 127 | 0.492 | 0.5517 | Yes |
| 41 | GMNN |  |  | 135 | 0.484 | 0.5542 | Yes |
| 42 | NCAPD2 |  |  | 136 | 0.483 | 0.5669 | Yes |
| 43 | TTK |  |  | 137 | 0.482 | 0.5796 | Yes |
| 44 | CDC45 |  |  | 154 | 0.456 | 0.5683 | Yes |
| 45 | BRIP1 |  |  | 168 | 0.443 | 0.5610 | Yes |
| 46 | MKI67 |  |  | 179 | 0.436 | 0.5579 | Yes |
| 47 | FEN1 |  |  | 180 | 0.435 | 0.5693 | Yes |
| 48 | CDC20 |  |  | 184 | 0.431 | 0.5763 | Yes |
| 49 | USP1 |  |  | 185 | 0.430 | 0.5876 | Yes |
| 50 | HMMR |  |  | 191 | 0.426 | 0.5915 | Yes |
| 51 | CKAP5 |  |  | 192 | 0.426 | 0.6027 | Yes |
| 52 | NEK2 |  |  | 194 | 0.423 | 0.6123 | Yes |
| 53 | MCM4 |  |  | 199 | 0.418 | 0.6175 | Yes |
| 54 | CCNE2 |  |  | 215 | 0.406 | 0.6063 | No |
| 55 | CDC6 |  |  | 239 | 0.388 | 0.5831 | No |
| 56 | PCNA |  |  | 242 | 0.386 | 0.5903 | No |
| 57 | POLA1 |  |  | 247 | 0.383 | 0.5945 | No |
| 58 | CCNB2 |  |  | 250 | 0.379 | 0.6016 | No |
| 59 | POLD3 |  |  | 252 | 0.377 | 0.6100 | No |
| 60 | GAS2L3 |  |  | 262 | 0.369 | 0.6066 | No |
| 61 | ANP32E |  |  | 280 | 0.358 | 0.5913 | No |
| 62 | DTL |  |  | 305 | 0.345 | 0.5654 | No |
| 63 | CDC25C |  |  | 307 | 0.343 | 0.5730 | No |
| 64 | RANGAP1 |  |  | 312 | 0.342 | 0.5762 | No |
| 65 | RFC2 |  |  | 331 | 0.333 | 0.5587 | No |
| 66 | UBR7 |  |  | 340 | 0.330 | 0.5558 | No |
| 67 | BLM |  |  | 408 | 0.305 | 0.4664 | No |
| 68 | DSCC1 |  |  | 422 | 0.299 | 0.4553 | No |
| 69 | G2E3 |  |  | 446 | 0.294 | 0.4296 | No |
| 70 | CBX5 |  |  | 457 | 0.291 | 0.4227 | No |
| 71 | UHRF1 |  |  | 652 | 0.255 | 0.1474 | No |
| 72 | RAD51 |  |  | 670 | 0.252 | 0.1294 | No |
Table: GSEA details [plain text format]

  

Fig 2: SEURAT\_CELLCYCLE: Random ES distribution      
 Gene set null distribution of ES for **SEURAT\_CELLCYCLE**
